# Alcohol use is associated with affective and interoceptive network alterations in bipolar disorder

**DOI:** 10.1002/brb3.2832

**Published:** 2022-11-30

**Authors:** Fiona M. Martyn, Genevieve McPhilemy, Leila Nabulsi, Jacqueline Quirke, Brian Hallahan, Colm McDonald, Dara M. Cannon

**Affiliations:** ^1^ Centre for Neuroimaging & Cognitive Genomics (NICOG), Clinical Neuroimaging Lab, NCBES Galway Neuroscience Centre, College of Medicine, Nursing, and Health Sciences National University of Ireland Galway Galway Galway H91 TK33 Ireland; ^2^ School of Psychology National University of Ireland Galway Ireland; ^3^ Imaging Genetics Center, Mark and Mary Stevens Neuroimaging & Informatics Institute University of Southern California Los Angeles California CA 90292 USA

**Keywords:** alcohol use, bipolar disorder, default mode network, executive control network, functional connectivity

## Abstract

**Introduction:**

Alcohol use in bipolar disorder (BD) is associated with mood lability and negative illness trajectory, while also impacting functional networks related to emotion, cognition, and introspection. The adverse impact of alcohol use in BD may be explained by its additive effects on these networks, thereby contributing to a poorer clinical outcome.

**Methods:**

Forty BD‐I (DSM‐IV‐TR) and 46 psychiatrically healthy controls underwent T1 and resting state functional MRI scanning and the Alcohol Use Disorders Identification Test‐Consumption (AUDIT‐C) to assess alcohol use. Functional images were decomposed using spatial independent component analysis into 14 resting state networks (RSN), which were examined for effect of alcohol use and diagnosis‐by‐alcohol use accounting for age, sex, and diagnosis.

**Results:**

Despite the groups consuming similar amounts of alcohol (BD: mean score ± SD 3.63 ± 3; HC 4.72 ± 3, *U* = 713, *p* = .07), for BD participants, greater alcohol use was associated with increased connectivity of the paracingulate gyrus within a default mode network (DMN) and reduced connectivity within an executive control network (ECN) relative to controls. Independently, greater alcohol use was associated with increased connectivity within an ECN and reduced connectivity within a DMN. A diagnosis of BD was associated with increased connectivity of a DMN and reduced connectivity of an ECN.

**Conclusion:**

Affective symptomatology in BD is suggested to arise from the aberrant functionality of networks subserving emotive, cognitive, and introspective processes. Taken together, our results suggest that during euthymic periods, alcohol can contribute to the weakening of emotional regulation and response, potentially explaining the increased lability of mood and vulnerability to relapse within the disorder.

## Graphical abstract

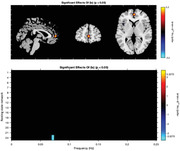

## 1 INTRODUCTION

Bipolar disorder (BD) is a chronic relapsing condition, characterized by fluctuations in mood state affecting approximately 1% of the world's population (Grande et al., 2016). Prevalence estimates of alcohol use disorders (AUD) in those with a diagnosis of BD are between 30% and 35%, suggesting that there is a significant proportion of this group who may consume alcohol below the clinical threshold for an AUD (Gordon‐smith et al., 2020). Moreover, those with a diagnosis of BD who do consume alcohol at non‐dependent levels are more likely to experience increased lability of mood than those who do not engage in alcohol use (Gordon‐smith et al., 2020; Goldstein et al., 2006). Most research examining the associations between resting state functional connectivity and alcohol use in BD has been undertaken in those with an AUD; however, this does not represent a complete spectrum of alcohol use by people with a diagnosis of BD (di Florio et al., 2014). While alcohol at the levels used most broadly in society may have some impact on functional connectivity (Table 1) (Hu et al., 2018), we hypothesize that its impact upon the already disrupted default, affective, and executive control networks (ECNs) in individuals with BD (Sha et al., 2018; Syan et al., 2018) may be additive thereby contributing to impaired mood stability and poorer clinical outcome in BD. Using a data driven model, this study will investigate the interactive effects of non‐dependent levels of alcohol use on intrinsic resting state networks (RSNs) in BD relative to psychiatrically healthy controls (HC).

**TABLE 1:** Studies examining the association of alcohol or Bipolar Disorder with functional connectivity

Authors	Sample size	Women:men	Age (years mean ± SD)	MRI data analysis methods	Assessment of alcohol use	Findings
**Non‐dependent alcohol use**						
Hu et al., 2018	N = 83	68:32	49 ± 19	Rs fMRI, SBA: amygdala	NIDA Quick Screen	↓ connectivity right dACC and amygdala, stronger relationship in men.
**Potential hazardous alcohol use**						
Vergara et al., 2017	N = 188	38:62	33 ± 9	Rs fMRI, ICA	AUDIT	↓ FC between SN, SM, precuneus, and visual networks. ↑ FC between reward and visual networks.
**Binge alcohol use**						
Arienzo et al., 2020	N = 35	54:46	25 ± 4	Rs fMRI, SBA	AUDIT	↑ connectivity between striatum, ACC, and OFC. ↓ connectivity between IFC and hippocampus.
Sousa et al., 2019	N = 34	52:48	20 ± 2	Rs fMRI, ICA	AUDIT	↑ FC in left ECN.
Herman, et al., 2018	N = 30	70:30	23 ± 5	Rs fMRI, ICA	Alcohol Use Questionnaire	↓ FC in VAT.
Crane et al., 2018	N = 46	21:89	26 ± 4	Rs fMRI, SBA: amygdala	Timeline Follow Back	↓ FC between right amygdala and OFC.
**Bipolar disorder** ^a^						
Syan et al., 2018	N = 23 studies	—	—	Review	Not asked	↔ within network alterations. Aberrant FC between amygdala, cingulate, medial, and ventrolateral prefrontal cortices.
Sha et al., 2018	N = 242 studies	41:59	29 ± 11	Review	Not asked	↓ FC in areas of SMN, DMN, DAN, VAT ↑ FC in areas of DMN, DAN, VAT, visual networks.
Vargas et al., 2013	N = 8 studies	—	—	Review	Not asked	Largest differences in FC within limbic‐striatal network
Du et al., 2015	N = 93	54:46	34 ± 11	Rs fMRI, ICA	Not asked	Insula cortex as discriminative region for BD.
Khadka et al., 2013	N = 374	53:47	38 ± 13	Rs fMRI ICA	Not asked	↑ FC in meso/paralimbic and ↓posterior DMN in patients. ↓ FC in fronto‐occipital, frontal/thalamic/basal ganglia, and ↑ SMN networks in patients and first‐degree relatives.
Meda et al., 2012	N = 374	53:47	38 ± 13	Rs fMRI ICA	Not asked	↑ FC between MPN and fronto‐temporal networks. ↓ FC between fronto‐occipital and anterior DMN/prefrontal.
Öngür et al., 2010	N = 46	43:57	39 ± 10	Rs fMRI, ICA: DMN	Not asked	↓ FC within mPFC, abnormal recruitment of parietal cortex.
Lois et al., 2014	N = 65	57:43	41 ± 9	Rs fMRI, ICA	Not asked	↑ FC between MPN and right FPN.

Abbreviations: AUDIT, Alcohol Use Disorders Identification Test; ACC, anterior cingulate cortex; BD, Bipolar disorder; B‐YAACQ, Brief Young Adult Alcohol Consequences Questionnaire; dACC, dorsal anterior cingulate cortex; DAN, dorsal attention network; DMN, default mode network; DMQ‐R SF, Drinking motive questionnaire revised short form; DRN, drinking only; ECN, executive control network; FC, functional connectivity; FPN, frontoparietal network; ICA, Independent component analysis; IFC, inferior frontal cortex; mPFC, medial prefrontal cortex; MPN, mesoparalimbic network; NIDA, National Institute on Drug Abuse; OFC, orbitofrontal cortex; PACS, Penn Alcohol Craving Scale; Rs fMRI, resting state functional magnetic resonance imaging; SBA, seed based analysis; SMN, sensorimotor network; SMAST, Short Michigan Alcohol Craving Scale; VAT, ventral attention network.

^a^BD participants were not comorbid for AUD.

### 1.1 Resting‐state connectivity in bipolar disorder

In BD, reduced resting‐state connectivity is demonstrated between the amygdala, prefrontal cortex, the anterior cingulate cortex (ACC), and the orbitofrontal cortex (OFC) in BD and is suggested to underlie mood dysregulation (Chase & Phillips, 2016; Meda et al., 2012). Alternatively, data driven approaches, such as independent component analysis (ICA) can be used to decompose patterns of intrinsic functional connectivity into discernible networks thus avoiding a spatially restricted approach introduced by the selection of seed regions of interest. Additionally, by utilizing BOLD spectral power analysis within ICA it is possible to investigate the spatial organization of brain oscillatory activity to describe with more depth the functional organization of the brain (Baria et al., 2011). Historically, preservation of intrinsic networks was suggested to be a feature of BD, with a review finding no difference in functional connectivity within networks decomposed through ICA (Table 1) (Syan et al., 2018). However, a recent meta‐analysis demonstrated increased and decreased functional connectivity within areas of the default mode network (DMN), sensorimotor, attention, and visual networks within the disorder (Sha et al., 2018). Dysconnectivity within and between default mode, salience, and cognitive networks modulating emotion processing is suggested to underlie symptoms of BD, with impairment in processing of emotional stimuli evident in the disorder (Lois & Linke, 2014). Moreover, altered functional connectivity between intrinsic networks has been demonstrated within the disorder, with increased connectivity between meso/paralimbic and fronto‐temporal and right frontoparietal networks (Meda et al., 2012; Lois & Linke, 2014). This increase in connectivity between networks subserving affective and interoceptive processing is additionally associated with increased negative symptoms, although usually not core symptoms of BD (Meda et al., 2012). Longitudinal imaging studies suggest compensatory and decompensatory activation patterns within frontolimbic networks which relate to illness phase and mood state (Lim et al., 2013). Taken together, this suggests that vulnerability to relapse within the disorder may be mediated by connectivity alterations within and between RSNs disrupting affective, cognitive, and introspective processes.

### 1.2 Resting‐state connectivity and alcohol use

Research on resting state connectivity in association with non‐dependent alcohol use is limited; however, reduced functional connectivity between the amygdala seed region and the right dorsal ACC has been demonstrated (Table 1) (Hu et al., 2018). Reduced functional connectivity in association with binge alcohol use is demonstrated between the amygdala and the OFC, as well as between the inferior frontal gyrus and hippocampus, areas involved in emotion and attention processes (Crane et al., 2018; Arienzo et al., 2020). Additionally, increased connectivity between striatal areas within the reward network and frontal areas involved in salience and attention networks are found in association with binge alcohol use (Arienzo et al., 2020). The increased connectivity demonstrated within the reward and salience networks are consistent with results found in AUD groups, which may point to a greater motivation to consume alcohol (Arienzo et al., 2020). Moreover, increased connectivity within nodes of the DMN was associated with increasing alcohol use in a sample of 25,378 UK residents; this study identified that the harms related to alcohol were more detrimental than those attributable to other modifiable factors (Topiwala et al., 2021). Recent studies within college‐aged samples have demonstrated increased connectivity in the left ECN in association with binge alcohol use and aberrant connectivity between the ventral attention network and right supramarginal gyrus (Sousa et al., 2019; Herman et al., 2019). Cycles of intoxication and withdrawal can lead to dysregulation of networks responsible for the effective processing of executive tasks, such as attention, inhibition, and goal‐orientated behavior (Volkow et al., 2004). Prior work has also demonstrated alterations to neural structure: Longitudinal samples have demonstrated that alcohol use is associated with reduced hippocampal volume and poorer cognitive outcomes in college‐aged students and older adults (Meda et al., 2019; Topiwala et al., 2017). In conjunction with the findings in BD samples, these studies suggest that alcohol use may place BD participants at additional vulnerability to aberrant connectivity within and between networks, particularly those associated with affective, cognitive, and introspective processing.

Alcohol use in BD is associated with mood lability and a poor clinical trajectory, and also with functional alterations to intrinsic RSNs subserving affective, cognitive, and introspective processes (Gordon‐smith et al., 2020; Goldstein et al., 2006; Sousa et al., 2019; Herman et al., 2019). We propose that non‐dependent alcohol use may place those with a diagnosis of BD at vulnerability to relapse through compound alterations to connectivity within and between functional networks. We expect a differential impact of alcohol use on intrinsic connectivity for those with a diagnosis of BD relative to controls, particularly in networks related to emotion, cognition, and introspection in addition to independent effects of use and of diagnosis on these core intrinsic networks’ functional connectivity.

## 2 METHODS

### 2.1 Participants

Participants to the study met with a registered psychiatrist for a Structured Clinical Interview (SCID), patient or control edition, and a diagnosis of BD type‐I or type‐II was confirmed using the DSM‐IV‐TR criteria (American Psychiatric Association 2000). All participants were between the ages of 18 and 65 years. Healthy control participants (HC) were admitted to the study if they had no DSM‐IV‐TR Axis‐I disorder, no first degree relative with a confirmed mental health diagnosis, and no current or historical use of psychotropic medication for the management of anxiety or mood disorders. Exclusionary criteria for all participants were: (i) a history of an AUD, (ii) a loss of consciousness lasting more than 5 min, (iii) pregnancy or breastfeeding, (iv) a current gastrointestinal disorder, and (v) heart problems, or uncontrolled blood pressure. All participants were sober and had not consumed alcohol prior to all of their study visits. All participants provided written consent; ethical approval was granted by the Galway University hospitals research ethics committee.

### 2.2 Assessment of alcohol use

Alcohol use data was collected via the Alcohol Use Disorders Identification Test‐Consumption (AUDIT‐Consumption, AUDIT‐C), which consists of the first three questions of the larger 10‐question AUDIT, and is validated for use in a variety of settings (Kristen et al., 1998). The scale measures frequency and amount of alcohol use over the previous 12 months, as well as the frequency of binge alcohol use occasions (≥ 6 standard drinks in one episode). Each item is scored from 0 to 4, with a maximum score of 12, while a score of > 5 on the AUDIT‐C indicates a potential for harmful use (Long & Mongan, 2013). A history of AUD excluded a participant from the study, and this assessment was made by a registered psychiatrist during a SCID.

### 2.3 Assessment of clinical signs and symptoms

The Hamilton Depression Rating Scale (HDRS) objectively and reliably quantifies depressive episodes (Hamilton, 1960). The scale is clinician rated and comprises 24 questions; scoring is based on the first 17 items, with a range of 0–53, a score of ≤ 8 indicates the absence of symptoms of a depressive episode (Hamilton, 1960). The Young Mania Rating Scale (YMRS) is an 11‐item scale which is valid and reliable for the identification of (hypo)manic episodes (Young & Meyer, 1978). Scoring is based upon objective ratings during a clinical interview, with a score of < 7 indicating the absence of (hypo)manic symptoms in the BD participants (Young & Meyer, 1978). Absence of symptoms related to depressive mood or (hypo)manic symptoms in the participants defines a participant as euthymic.

### 2.4 MRI acquisition

MRI data for all participants were obtained using a high‐resolution 3T Achieva scanner (Philips Medical Systems, Netherlands) at the Wellcome Trust Health Research Board Centre for Advanced Medical Imaging (CAMI), at St James's Hospital, Dublin, Ireland. A high‐resolution 3‐dimensional structural T1‐weighted Magnetization Prepared Rapid Acquisition Gradient Echo sequence was acquired using an 8‐channel head coil (echo time [TE] 3.9 ms; repetition time [TR] 8.5 ms; flip angle 8°; 1 mm^3^ isotropic voxel size, 180 slices). Resting state data were acquired using a single shot gradient echo planar imaging sequence, involving whole‐brain acquisition of 180 volumes (repetition/echo times = 2000/28 ms, flip angle 90^°^, field of view 240 × 240 × 133 mm, 3 × 3 mm voxel dimensions, 80×80 matrix size, and 38 axial slices of 3.2 mm each). Resting state scans were acquired while participants were supine with eyes open and instructed to remain fixed on a crosshair for the 6‐min scan duration.

### 2.5 Subject‐level image preprocessing

Motion correction of MR images were undertaken using a six‐parameter rigid body transform and interpolated using a fourth‐degree b‐spline. Corrected images were inspected to assess translation and rotation parameters; all motion correction was within the bound of one voxel, with no spikes in motion greater than half a voxel. Within subject co‐registration of structural to functional images was undertaken using a rigid‐body model and image reslicing. Images were again inspected to ensure anatomically accurate alignment for each participant. Structural images were segmented to create maps of gray, white, and CSF tissue types, with a bias corrected image created. This image was then spatially normalized using affine transformations. Functional and structural images were normalized to the Montreal Neurological Institute (MNI) template. Images were spatially smoothed with a Gaussian kernel of 6 mm at full width half maximum. Pre‐processing of fMRI data was undertaken using Statistical Parametric (SPM12) software (Wellcome Centre for Human Neuroimaging) (Friston et al., 1994).

### 2.6 Group‐level spatial independent component analysis

A model order of 30 was chosen to ensure spatial stability of derived components with RSNs replicated in literature (Abou‐Elseoud et al., 2010). Group component extraction was undertaken using the Infomax algorithm, which was repeated 20 times in ICASSO to maximize the stability of the decomposed components (Jafri et al., 2008). Group level IC's were back reconstructed using GIG‐ICA, a method which obtains subject‐specific ICs with stronger independence and spatial correspondence across subjects, as well as increased accuracy (Du & Fan, 2013). All steps related to spatial ICA were undertaken using the GIFT Toolbox (Calhoun et al., 2001).

### 2.7 Selection of RSNs

Two researchers (FMM and JQ) independently assessed each IC to classify the components as likely signal related to RSNs or noise related to motion, physiological processes, or low signal drift. A component was classified as an RSN if it displayed a low number of large clusters with peaks of activation located in the gray matter, had a regularity of time course oscillations with power spectra in the low‐frequency range, was spatially correlated with gray matter templates in SPM8, and had a higher ratio of gray matter in comparison to white matter and cerebrospinal fluid (Griffanti et al., 2017). Potential networks were cross referenced with networks which have been replicated in the literature (Laird et al., 2011). Neuroanatomical locations of significant signal changes were obtained by overlaying masks of the RSN images onto the MNI template. The Harvard‐Oxford cortical and subcortical atlas was used to label regions based on cytoarchitectonic probabilities, which was accessed through the SPM Anatomy toolbox (Eickhoff et al., 2005).

### 2.8 Functional connectivity and statistical analysis

We analyzed three distinct but complementary aspects of functional connectivity to describe within and between‐network connectivity in relation to alcohol and diagnosis, these being: spatial map intensity, BOLD power spectra, and functional network connectivity (Allen et al., 2011). The spatial maps of each RSN were analyzed to identify differences in the participation of a voxel or cluster of voxels in a network which relates to the level of connectivity and degree of coactivation within a network (Allen et al., 2011). The power spectra of RSN timecourses relating to the level of coherent activity at a specific frequency within a network were analyzed by estimating each BOLD spectrum based on detrended subject‐specific timecourses, using a multi‐taper approach: a bandwidth of three and number of tapers set to five using the Mancovan toolbox (Allen et al., 2011). Functional network connectivity relates to the connectivity between networks of interest; timecourses of ICA‐created RSNs were band‐pass filtered with a Butterworth filter with cutoff frequencies of .008–1.5 Hz to maximize the likelihood of signal related to RSNs and reduce the likelihood of signal related to motion and physiological processes (Luchtmann et al., 2010). Relationships between spatial map intensities, BOLD spectral power, functional network connectivity, and independent variables: diagnosis, AUDIT‐C score, and an interaction between diagnosis and AUDIT‐C score, controlling for age, sex, and motion parameters were assessed using the Mancovan toolbox in GIFT. An alpha level of .05 was used for all analyzes, with results corrected for multiple comparison using the false discovery rate (FDR). Group differences in demographic and clinical data were assessed using Chi‐squared for categorical data (sex, hazardous use, and binge alcohol use), and a *t*‐test for normally distributed data (age). Non‐normally distributed data were assessed using the Mann–Whitney U (AUDIT‐C, Hollingshead Scale, HDRS, TMRS). Statistical significance was assessed using a two‐tailed *α* level of .05 (SPSS s v.24 IBM Corp., New York, USA).

## 3 RESULTS

### 3.1 Sample demographic and clinical characteristics

A total of 86 individuals participated in this study, 40 participants with a diagnosis of BD (33 BD‐I, 7 BD‐II) and 46 HC. As the groups were matched for age and sex, no significant differences were found (Table 2). A significant difference was found between the groups in scores on the HDRS (*U* = 339.5, *p <* .000), and the YMRS (*U* = 678.5, *p* = .016). The majority of the BD participants were euthymic at the time of scanning (*n* = 28, 70%). A statistically significant difference was found in socioeconomic status, with BD participants more likely to report a lower status in comparison to control participants (*U* = 583.5, *p* = .004).

**TABLE 2:** Clinical and demographic characteristics of the sample

	Healthy controls n = 46	Bipolar participants n = 40	Statistical comparison Test statistic, p
Sex (f:m, n)	30:16	22:18	χ^2^ = 0.934, .381
Age at MRI (years)	41.00 ± 14	43.08 ± 13	T = ‐0.721, .473
SES status (mean ± SD)	42.22 ± 16	31.52 ± 16	U = 583.5, .004*
HDRS (mean ± SD)	1.04 ± 1.7	6.78 ± 6.6	U = 339.5, .000*
YMRS (mean ± SD)	0.72 ± 1.5	1.80 ± 2.6	U = 678.5, .016*
Lithium use (no, %)	0, 0	26, 65	—
Antipsychotic use (no, %)	0, 0	13, 33	—

*Note*: Socioeconomic status was assessed using the Hollingshead Scale. Data are presented as mean ±sd.

Abbrevations: f, female; HDRS, Hamilton Depression Rating Scale; m, male; YMRS, Young Mania Rating Scale.

^*^significant at α = .05.

### 3.2 Comparable alcohol use scores between the groups

There was no difference between the diagnostic groups in alcohol use scores (Table 3; Figure S1). The two groups did not differ significantly in the reporting of their frequency of engaging in binge drinking episodes, (i.e., consuming six or more alcoholic drinks in one sitting) (Table 2) (Kristen et al., 1998). The reported AUDIT‐C scores represent a wide range of non‐normally distributed alcohol use scores within our participants, ranging from no alcohol use (a score of 0) to a potential for consuming alcohol daily or almost daily (maximum score 11). There was no association between alcohol use and SES scores for HC (*r* = ‐.164, *p* = .277) or BD (*r* = .087, *p* = .593).

**TABLE 3:** No difference in alcohol use scores between the groups

	Healthy controls n = 46	Bipolar participants n = 40	Statistical comparison Test statistic, p
AUDIT‐C (mean ± SD)	4.74 ± 2.81	3.63 ± 3	U = 713.5, p = .072
Positive for hazardous drinking (n,%)^a^	21 (46)	14 (35)	χ2 = 1.006, p = .316
Frequency of binge (n)^b^	Never: 15 Less than monthly: 16 Monthly: 5 Weekly: 10	Never: 19 Less than monthly: 11 Monthly: 5 Weekly: 5	χ2 = 2.658, p = .447

^a^ Harmful use is defined as scoring >5 in the AUDIT‐C.

^b^ A binge is defined as drinking more than six standard drinks in one setting.

Abbreviation: AUDIT‐C, Alcohol Use Disorders Identification Test‐Consumption.

### 3.3 Resting state networks within the data

Following decomposition into 30 networks, one ICN (30) was not retained due to low stability analysis as determined by the cluster quality index, *I*_q_ > .938 (Himberg et al., 2004). After manual classification, 16 ICNs were deemed noise as activation occurred within areas of white matter and cerebral spinal fluid space, time courses were irregular, results displayed power spectra within frequency bands aligned with cardiac pulsation or respiratory noise, or it had low correlation with gray matter templates in SPM8. Of the remaining 14 components (Figure S2), correspondence with templates from Shirer *et al.* (2012) was established to describe the RSNs anatomically as: dorsal DMN (RSNs 6, 12, 21), ventral DMN (RSNs 7, 13), anterior salience (RSNs 4, 9, 16), executive control (RSNs 8, 24), sensorimotor (RSNs 1, 19, 20), and auditory networks (RSN 18).

### 3.4 Altered resting state connectivity in relation to alcohol use and a diagnosis of bipolar disorder

**FIGURE 1:**
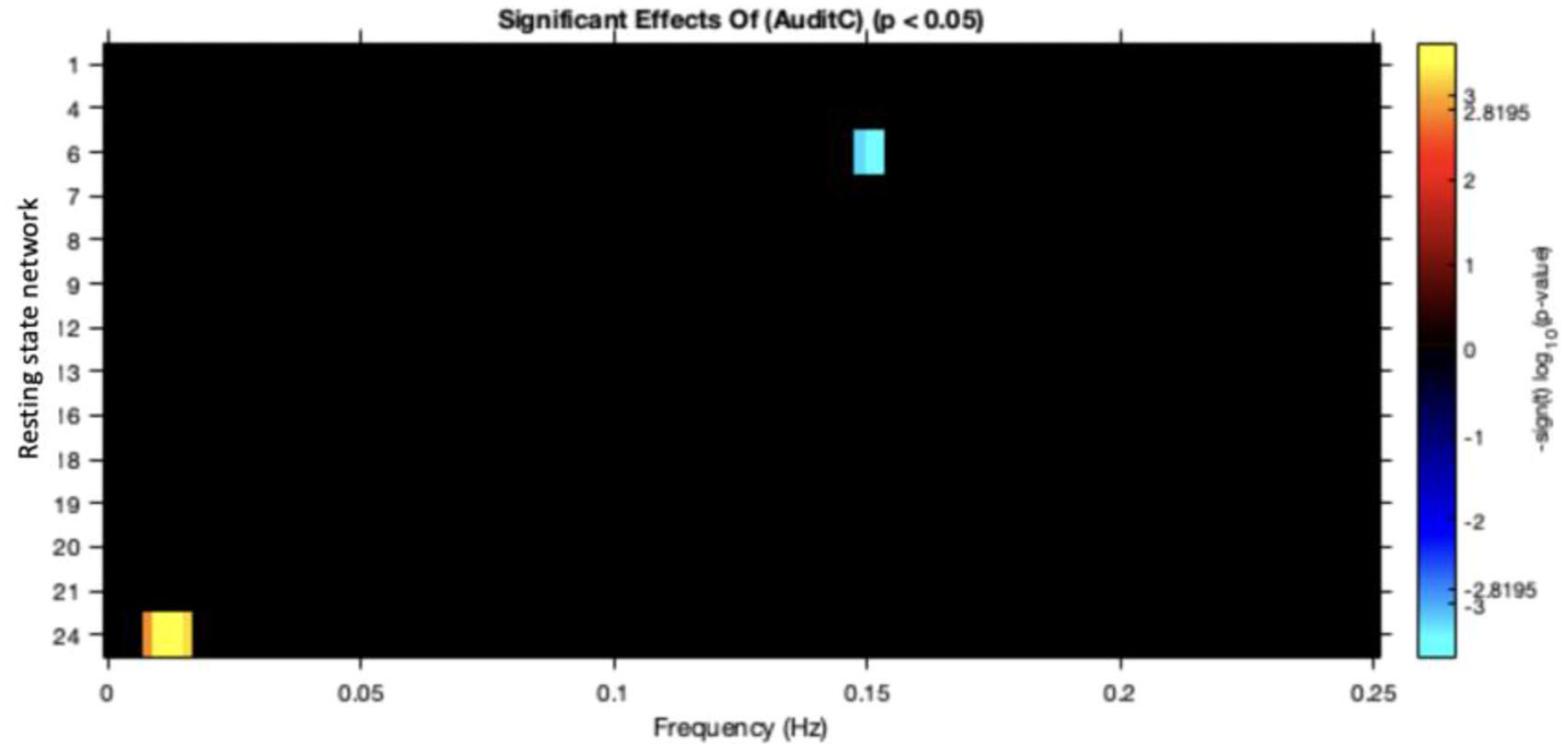
Alcohol is significantly associated with altered connectivity within default mode and executive control networks. In all figures, red/orange indicates a positive relationship and blue a negative; *p*‐values are corrected using the false discovery rate. DMN, default mode network; RSN, resting state network.

For all participants within this study (HC and BD), within network connectivity or between network connectivity was not associated with alcohol use only for all 14 RSN. However, spectral analysis demonstrated that greater alcohol use was associated with reduced BOLD spectral power at 0.15 Hz in a dorsal DMN network and greater BOLD spectral power within the low frequency range (> 0.05 Hz) in an ECN (Figure 1) (all *p‐*values FDR corrected).

**FIGURE 2:**
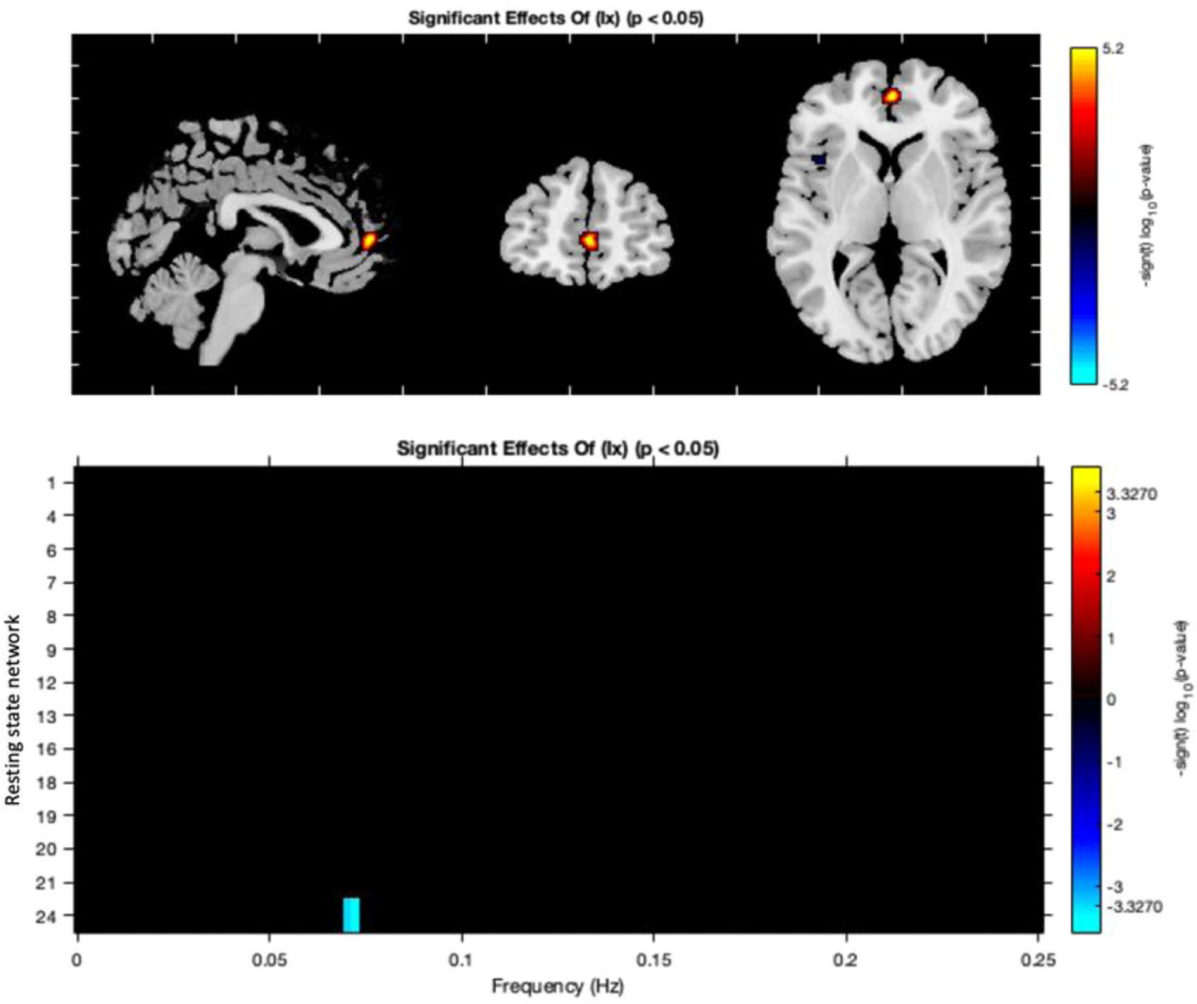
A diagnosis of bipolar disorder is significantly associated with altered connectivity within default mode and executive control networks. In all figures, red/ orange indicates a positive relationship and blue a negative; *p*‐values corrected using the false discovery rate. DMN, default mode network; RSN, resting state network

A between group test found that for participants with a diagnosis of BD, greater alcohol use was associated with a statistically significant increase in connectivity of the paracingulate cortex within a dorsal DMN (RSN 12, Figure 2; Figure S3). A significant decrease in BOLD spectral power between 0.05 and 0.1 Hz within an ECN (RSN 24) was also detected (Figure 2). In contrast, connectivity between the examined networks did not relate to alcohol consumption in HC or BD groups.

**FIGURE 3:**
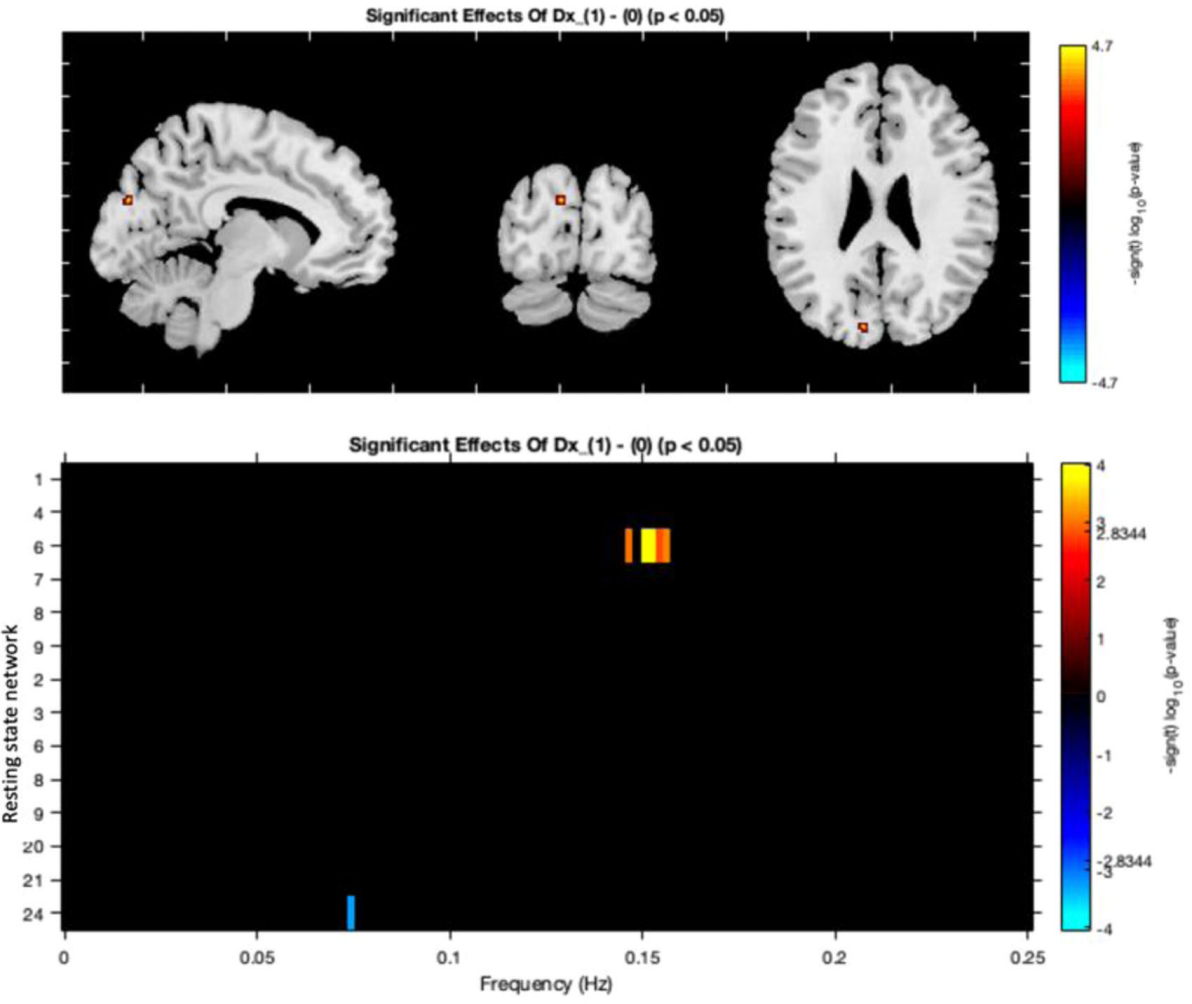
A diagnosis of bipolar disorder with increasing alcohol use is significantly associated with altered connectivity within default mode and executive control networks. In all figures, red/ orange indicates a positive relationship and blue a negative; *p*‐values corrected using the false discovery rate. DMN, default mode network; RSN, resting state network

A within‐group test found that a diagnosis of BD was associated with a statistically significant increased connectivity of the cuneal cortex within the ventral DMN (RSN 13) (Figure 3). Additionally, a significant decrease in BOLD spectral power within an ECN (RSN 24) between 0.05 and 0.1 Hz was detected for participants with a diagnosis of BD, with greater BOLD spectral power within a dorsal DMN (RSN 6) at 0.15 Hz (Figure 3). A diagnosis of BD did not relate to connectivity between the examined networks.

## 4 DISCUSSION

Using the data‐driven multivariate approach of ICA, we sought to determine the impact of alcohol on intrinsic RSNs and if there was a differential impact for people with a diagnosis of BD. We demonstrate that for all participants in the study (HC and BD), increasing alcohol consumption was associated with alterations to the level of coherent activity at specific frequencies within default mode and ECNs. Additionally, we find that having a diagnosis of BD, but moreover having a diagnosis of BD and consuming alcohol is significantly associated with increased connectivity within default mode networks and reduced spectral power at 0.05–0.1 Hz in an executive control network. These results suggest that introspective and cognitive networks are impacted by alcohol, over and above BD alone. Altered functionality related to emotion processing subserved by these networks may contribute to vulnerability to relapse within the disorder.

### 4.1 Measuring the spatial organization of the brain

Functional connectivity studies typically characterize connectivity by the degree of coactivation within a network or by the connectivity between networks; however, by investigating the spatial organization of brain oscillatory activity, it may be possible to describe with more depth the functional organization of the brain (Baria et al., 2011). Unimodal brain areas which encode basic features of sensation transmit information to transmodal limbic or paralimbic areas (Mesulam, 1998). This increase in complexity corresponds to changes in power distribution where unimodal areas are dominated by low‐frequency power and transmodal by high‐frequency power distributions (Baria et al., 2011). We demonstrate that for participants with BD who engage in alcohol use, there is a reduction in the coherence of oscillatory activity between 0.05 and 0.1 Hz within an ECN. This may reflect reduced engagement of the network at a particular frequency or a compensatory mechanism in association with alcohol use within the disorder. Previous work has demonstrated that in comparison to control participants, patients with a diagnosed mood disorder displayed less low‐frequency spectral power in a dorsal DMN, which was associated with life‐long suicidal ideation (Malhi et al., 2020). These results suggest that a depressed state may be associated with a pattern that differs from what we would have detected in our primarily euthymic sample. Taken together, they demonstrate the relevance of investigating BOLD power spectra in higher‐order mood disorders to better understand the spatial organization of brain oscillatory activity.

The brain's functional network is underpinned by structural or anatomical architecture; the local and distributed topologies of these networks constrain function while allowing for adaptability to environmental stimuli (Petersen & Sporns, 2015). The relational architecture of these networks facilitates the emergence of cognitive processing; distributed and interactive mapping of anatomy onto computation supports a degenerative system and adaptability of function (Mesulam, 1990). Low levels of alcohol use are found to impact on neurovascular coupling leading to complex alterations and differential activation patterns across cortical regions (Bressler & Tognoli, 2006). Correspondence between large‐scale intrinsic networks and behavior is consistent across the literature; however, the requirement for degeneracy and adaptability of function means that coordinated patterns within local area networks can change dynamically (Bressler & Tognoli, 2006). These dynamic patterns of local activity give rise to large‐scale intrinsic networks (Bressler & Menon, 2010). Suggesting that function is dependent on the smooth interactions of local and large‐scale networks, dysfunction at either scale can point to individual differences within the system (Luchtmann et al., 2010).

### 4.2 Alcohol and functional connectivity

We demonstrate for all participants in this study, in the presence of increasing alcohol use, there was a reduction in the coherence of oscillatory activity at 0.15 Hz in a DMN and a greater coherence of oscillatory activity at a low frequency (> 0.5 Hz) in an ECN. A recent large‐scale prospective cohort study demonstrated increased functional connectivity within the DMN in a dose‐dependent manner (Topiwala et al., 2021). The average alcohol intake for this sample was 13.5 standard alcoholic drinks per week. Despite this being in keeping with current UK government guidelines, the results suggest that even low levels of alcohol use are harmful to the brain. Our demonstrated results in the DMN support these findings, while expanding on them to include alterations in a cognitive network. Previous work by Vergara et al. (mean AUDIT score = 15.3 ± 5.3) and Sousa et al. (11.2 ± 3.25) report functional alterations within and between networks supporting cognitive and emotional processes, which supports our results in an ECN. The studies by Vergara et al. and Sousa et al. report AUDIT scores which are significantly higher than the average score for our sample (5.45 ± 4.9). Together with the recent work by Topiwala et al., this suggests that functional alterations of intrinsic networks are apparent at a range of alcohol consumptions which are common in our communities. The impact of alcohol on the brain depends on numerous molecular mechanisms, which in turn can be reflected in differences of activation as measured by the BOLD response (Luchtmann et al., 2010). Previous work has demonstrated that a low amount of alcohol does not distort simple repetitive movements but suggests that more complex movements or behaviors may be impacted due to the decrease of neuronal activation, as well as the potential for the loss of neurovascular coupling due to alcohol‐induced vasodilation (Luchtmann et al., 2010). Moreover, it suggests that circuitry involved in affective or cognitive functions may be more susceptible to alcohol (Luchtmann et al., 2010), supporting the findings of this study that non‐dependent alcohol use impacts activation coherence in cognitive and introspective networks.

### 4.3 Functional connectivity in bipolar disorder with alcohol use

We found increased connectivity of the paracingulate cortex in a dorsal DMN and reduced coherence of oscillatory activity at 0.5–1.0 Hz within an ECN for participants with a diagnosis of BD who consumed alcohol, in comparison to HC. Moreover, for the paracingulate gyrus, increasing connectivity is related to increasing alcohol use for BD participants only, demonstrating diagnosis‐specific associations with alcohol. Pre‐existing alterations in structure and function of the cingulate are associated with difficulties in emotional processing in BD (Jabbi et al., 2020; Comte et al., 2016), This suggests that the compound impacts of diagnosis and alcohol use may place further stress on network function, particularly relating to affective and introspective behaviors. The DMN comprises a large set of co‐activated brain areas that form a system for self‐monitoring, autobiographical thought, and perceiving the perspectives of others (Buckner et al., 2008). The ECN is proposed to be responsible for high‐level cognitive processes, for instance orientating attention and identifying emotional stimuli (Bressler & Menon, 2010). The DMN and ECN engage in discrete cognitive processes and guide responses to emotionally salient stimuli (Bressler & Menon, 2010). A recent meta‐analysis demonstrated reduced within‐network connectivity of a network corresponding to our ECN for patients in an acute mood state in contrast to those with the disorder in a remitted state (Wang et al., 2020). These reductions in connectivity in the ECN are suggested to reflect instabilities of mood which are prominent within the disorder, with a normalization of connectivity in the remitted state reflecting improved function and affective processing (Wang et al., 2020). We demonstrated reduced connectivity within the ECN for those with a diagnosis of BD who are predominately euthymic while controlling for alcohol use within the sample. The lack of control for confounding factors may have influenced the findings of Wang et al. (2020) and suggests that alcohol may be an important factor which has not been adequately controlled for in previous research. We have previously demonstrated an effect of alcohol use on the topological configuration of an anatomical subnetwork, including areas that are involved in introspective and affective functions for BD participants only (Martyn et al., 2021). We suggest that this reflects a diagnosis‐specific biological vulnerability, which may be associated with the deleterious illness course of BD when associated with alcohol use (Martyn et al., 2021). These previous findings support our results of compound alterations within the ECN for those with a diagnosis of BD in the presence of increasing alcohol use. We suggest that the compound effect of alcohol consumption and a diagnosis of BD impacts on the coherence of oscillatory activity within this RSN, potentially weakening emotional control during euthymic periods. Changes in functional connectivity, as demonstrated by reduced BOLD spectral power of the ECN, combined with increased connectivity within the DMN may point to vulnerability in identifying and responding to emotionally salient internal and external stimuli in BD in association with alcohol use, thus impacting on vulnerability to increased mood lability in the disorder.

### 4.4 Functional connectivity in bipolar disorder

We demonstrate increased functional connectivity of the cuneal cortex and greater coherence of oscillatory activity at 0.15 Hz within a DMN and reduced coherence of oscillatory activity at 0.05–1.0 Hz within an ECN in association with a diagnosis of BD. Reduced functional connectivity within the DMN during acute mood episodes in contrast to hyperconnectivity within DMN in remitted mood states has been previously reported in BD relative to HC (Wang et al., 2020). Alterations of connectivity within the DMN are suggested to reflect heightened planning in relation to internal and external environments for those with a diagnosis of BD (Lois & Linke, 2014). The majority of our sample was euthymic at the time of scanning, and our results support this distinction in DMN connectivity. Moreover, alterations of decreased connectivity within a ventral DMN have been demonstrated within BD and for their first‐degree relatives (Khadka et al., 2013); our results support changes within the connectivity of a ventral DMN within BD and provide evidence for functional alterations, which may impact interoceptive processes within the disorder. The DMN can be subdivided into regions and while this may be a function of dimension reduction, frequency of oscillatory activity within the DMN is demonstrated to change from low frequency in posterior to high frequency in anterior portions (Baria et al., 2011). This points to a functional heterogeneity within the network and challenges the idea that the entire network is suppressed in association with task demands (Buckner et al., 2008). Our results identify ventral and dorsal components of the DMN and demonstrate alterations to within network connectivity for these subcomponents of the network. The successful suppression of the DMN is critical for cognitive operations, such as attentional and emotional response, processes which are known to be compromised within BD (Buckner et al., 2008). Our demonstrated increase in DMN activation may reflect difficulties in suppression and orientation toward cognitively controlled emotional regulation, leading to the exacerbation of mood dysregulation seen within the disorder.

We have demonstrated alterations in power spectra within dorsal DMN and an ECN for people with a diagnosis of BD. Low‐frequency oscillations have been used to identify large‐scale networks and to study their organizational properties (Luchtmann et al., 2010). The largest power spectra for the DMN is located within the low frequency, with the posterior portion of the DMN dominated by the low frequency, and frontal portions showing oscillations at higher frequencies, with a suggestion that areas of the brain responsible for more complex processes are dominated by higher frequencies (Baria et al., 2011). Within our ECN is the insula, a structure which supports switching between the DMN and the ECN, so that attention is orientated toward cognitively controlled states (Bressler & Menon, 2010). Participants with a diagnosis of BD demonstrate deficiencies in switching from internally focused processes to task‐related processing in the presence of cognitive‐affective stimuli (Ellard et al., 2019). This occurs through increased activation within the DMN and reduced activation of the insula, suggesting that BD participants are less able to disengage from self‐monitoring processes in the presence of emotional distractors and move to a cognitively controlled state (Ellard et al., 2019). Our findings of increased DMN activation and reduced power of activation within an ECN support these findings, suggesting that the coherent activity of the networks is disrupted within the disorder, and may point to network vulnerability to relapse, possibly exasperated by the consumption of alcohol. The reductions in coherent activation within intrinsic networks suggest that brain states reflect dynamics, whereby interactions between frequency oscillators are critical aspects of these dynamics, which are embedded within the anatomical structure of the brain. This then reflects the need to approach fMRI or connectivity from a dynamic perspective, rather than single point estimate of function. Observing energy requirements to transitions between brain states and the alterations that may be present in BD in association with alcohol use will provide a richer explanation of the dynamic system of the brain within the disorder. A challenge to studying BD is the intricate and diffuse symptoms reported among patient groups. The use of data‐driven approaches can be beneficial as they analyze whole brain function and can identify weak contributions from numerous regions which may point to specific‐disorder‐related pathology (Calhoun, 2018). Applying this multivariate method may provide more sensitive evidence of the impact of alcohol use in BD and its contribution to mood lability and relapse within the disorder and contribute to the identification of mechanisms related to the disorder. This work contributes to previous knowledge by pointing to specific network patterning that is altered within the disorder, which may influence the regulation of interoceptive processes and the engagement of networks supporting cognitively controlled emotional processing.

### 4.5 Limitations to the study

The interpretation of RSNs is challenging as there is currently no global agreement on naming conventions or dimension reduction for networks. We chose to decompose our data to 30 components with the aim of aligning the spatial correspondence between our networks and those reliably replicated in the literature (Abou‐Elseoud et al., 2010). This model order was a trade‐off between large scale and finer grained network components, as we were interested in networks which often appear at the finer level, for example, the salience network and also large‐scale networks, that of the DMN. Resting state connectivity was initially identified at low frequency; however, RSN activity has been identified at frequencies as high as 1.5 Hz (Gohel & Biswal, 2015). The majority of our understanding of resting‐state signal lies within the lower frequency; therefore, our results of altered coherence within oscillatory activity at 1.5 Hz require further work to clarify its application to cognitive and affective networks. A limitation to our study is that alcohol use has been recorded as a self‐report measure, with no collateral attained to corroborate the quantity of alcohol use. However, the AUDIT‐C has been validated for use across a variety of setting and is a well‐used tool in research studies (Kristen et al., 1998). Additionally, in clinical settings, alcohol consumption is collected via self‐report, giving our results some measure of clinical relevance.

## 5 CONCLUSION

In a first endeavor to understand the functional impact of non‐dependent alcohol use in BD, we demonstrate alterations to within‐network connectivity of interoceptive and cognitive networks, which are involved in the regulation of emotion. Despite the groups consuming comparable amount of alcohol, we demonstrate alterations to default mode and ECNs for people with a diagnosis of BD, relative to HC. This alteration may impact on previously identified deficiencies in switching from internally to externally focused tasks during emotion processing, which results in a lack of ability to disengage in self‐monitoring processes in BD (Ellard et al., 2019). These alterations within network connectivity suggest that even during euthymic periods, alcohol can contribute to the weakening of emotional regulation and response, potentially explaining the increased lability of mood and vulnerability to relapse within the disorder.

## CONFLICT OF INTEREST

The authors report no biomedical financial interests or potential conflicts of interest.

### PEER REVIEW

The peer review history for this article is available at https://publons.com/publon/10.1002/brb3.2832

## Supporting information


**Supplementary Figure 1**. No Difference in Alcohol Use Scores Between the Groups
*Legend*: BD: Bipolar disorder; HC: Healthy control.

**Supplementary Figure 2**. Resting State Networks Obtained Through Independent Component Analysis
L*egend*: Resting state networks derived from spatial ICA are overlaid on a canonical brain thresholded at false discovery rate *p*<0.05. RSN number is displayed beneath, network classification is derived from Shirer *et al.*. DMN: default mode network; RSN: resting state network. Activation thresholded at *T*>20.

**Supplementary Figure 3**. Functional activation of the paracingulate gyrus is increased for those with a diagnosis of bipolar disorder who consume alcohol. *Legend*: BD: participants with a diagnosis of bipolar disorder; HC: healthy controls

## Data Availability

Data may be available for sharing, please contact dara.cannon@nuigalway.ie to discuss opportunities.
