# Supplementary material for: Alcohol use is associated with affective and interoceptive network alterations in bipolar disorder
Source: Brain Behav. 2022 Nov 30;13(1):e2832. doi: 10.1002/brb3.2832 (PMC9847622; doi:10.1002/brb3.2832)
Supplement: Supplementary file 1 — Supplementary Figure 1. No Difference in Alcohol Use Scores Between the Groups Legend: BD: Bipolar disorder; HC: Healthy control. [file BRB3-13-e2832-s003.pdf]

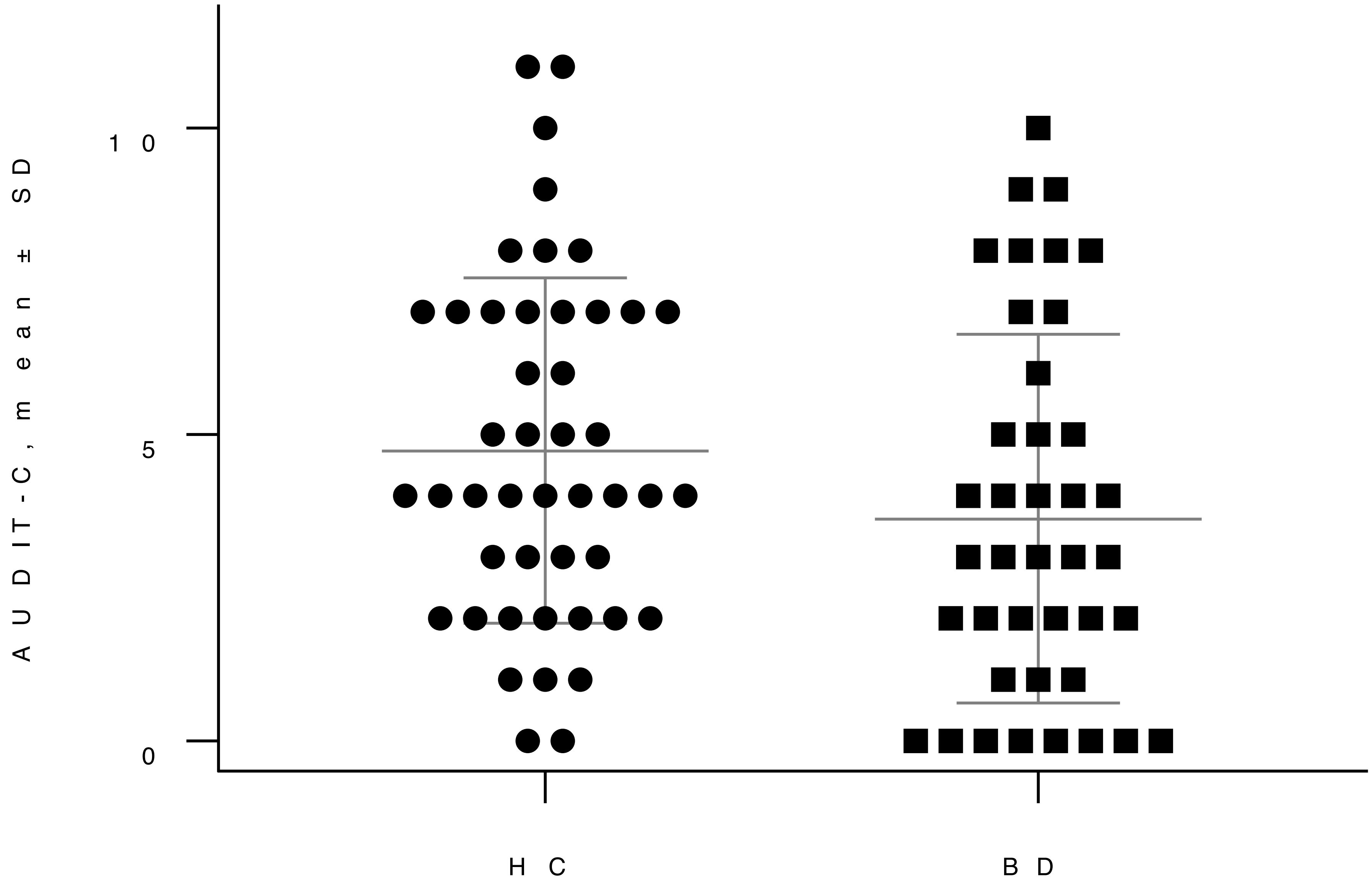
AUDIT-C, mean ± SD
10
5
0
HC
BD

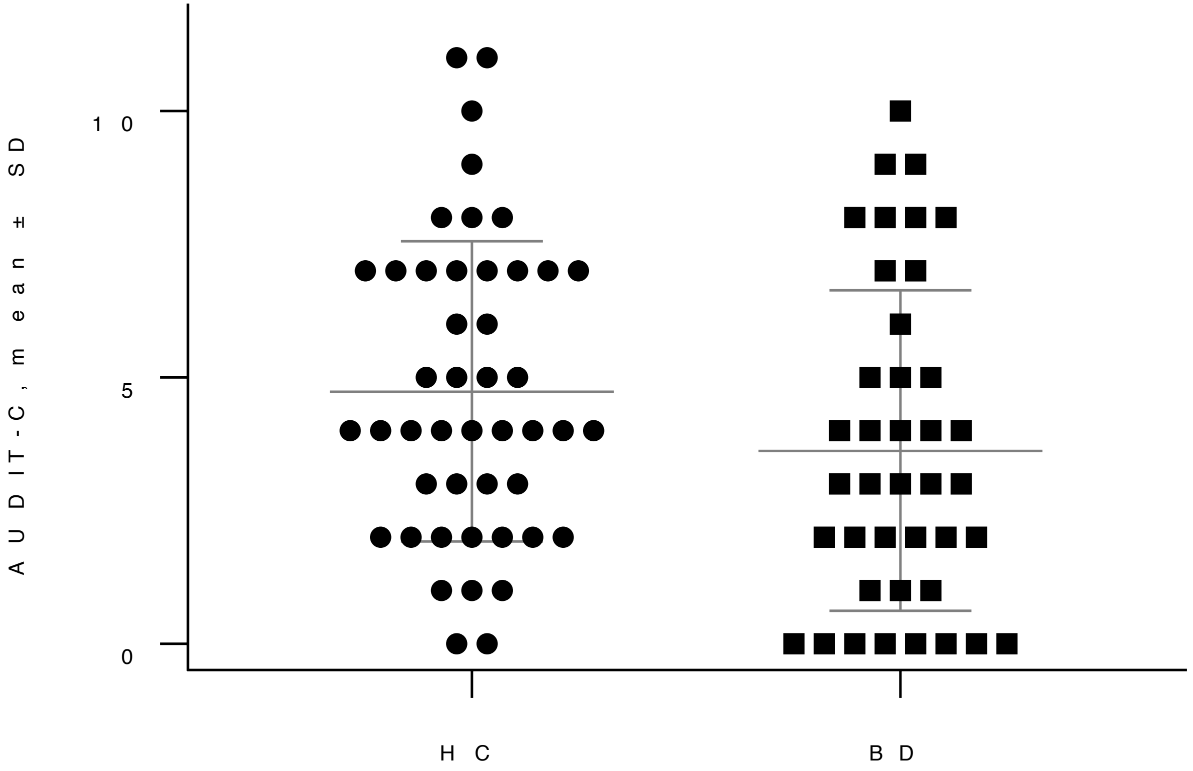

	Healthy controls<br>n=46	Bipolar participants<br>n=40	Statistical comparison<br>Test statistic, *p*
AUDIT-C (mean±SD)	4.74±2.81	3.63±3	U=713.5, p= 0.072
Positive for hazardous drinking (n,%) †	21 (46)	14 (35)	χ2=1.548, p= 0.316
Frequency of binge (n) ‡	Never: 15<br>Less than Monthly: 16<br>Monthly: 5<br>Weekly: 10	Never: 19<br>Less than Monthly: 11<br>Monthly: 5<br>Weekly: 5	χ2=2.658, p= 0.447

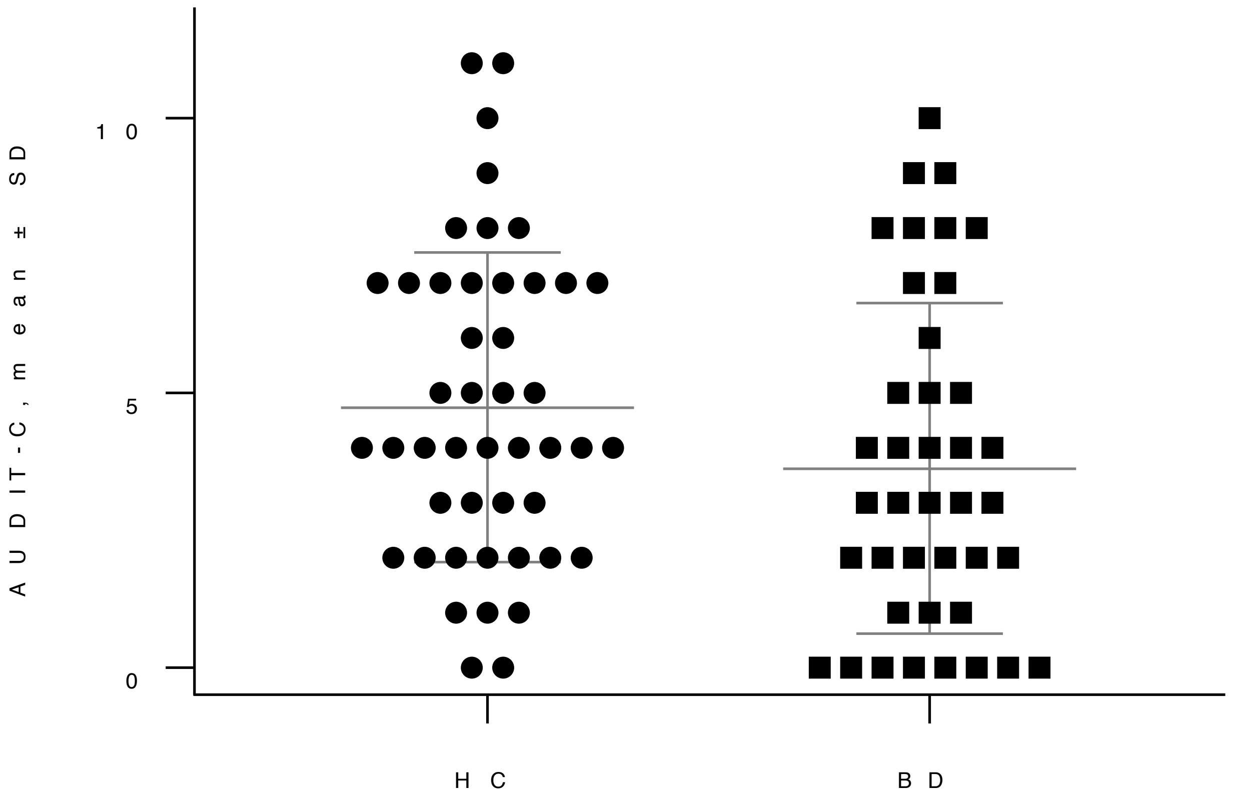

	Healthy controls n=46	Bipolar participants n=40	Statistical comparison Test statistic, *p*
AUDIT-C (mean±SD)	4.74±2.81	3.63±3.01	U=713.5, p= 0.072
Positive for hazardous drinking (n,%) †	21 (46)	14 (35)	χ2=1.548, p= 0.316
Frequency of binge (n) ‡	Never: 15<br>Less than Monthly: 16<br>Monthly: 5<br>Weekly: 10	Never: 19<br>Less than Monthly: 11<br>Monthly: 5<br>Weekly: 5	χ2=2.658, p= 0.447
