# Supplementary material for: Alcohol use is associated with affective and interoceptive network alterations in bipolar disorder
Source: Brain Behav. 2022 Nov 30;13(1):e2832. doi: 10.1002/brb3.2832 (PMC9847622; doi:10.1002/brb3.2832)
Supplement: Supplementary file 2 — Supplementary Figure 2. Resting State Networks Obtained Through Independent Component Analysis Legend: Resting state networks derived from spatial ICA are overlaid on a canonical brain thresholded at false discovery rate p<0.05. RSN number is displayed beneath, network classification is derived from Shirer et al.. DMN: default mode network; RSN: resting state network. Activation thresholded at T>20. [file BRB3-13-e2832-s002.docx]

**Supplementary Figure 2.** Resting State Networks Obtained Through Independent Component Analysis *
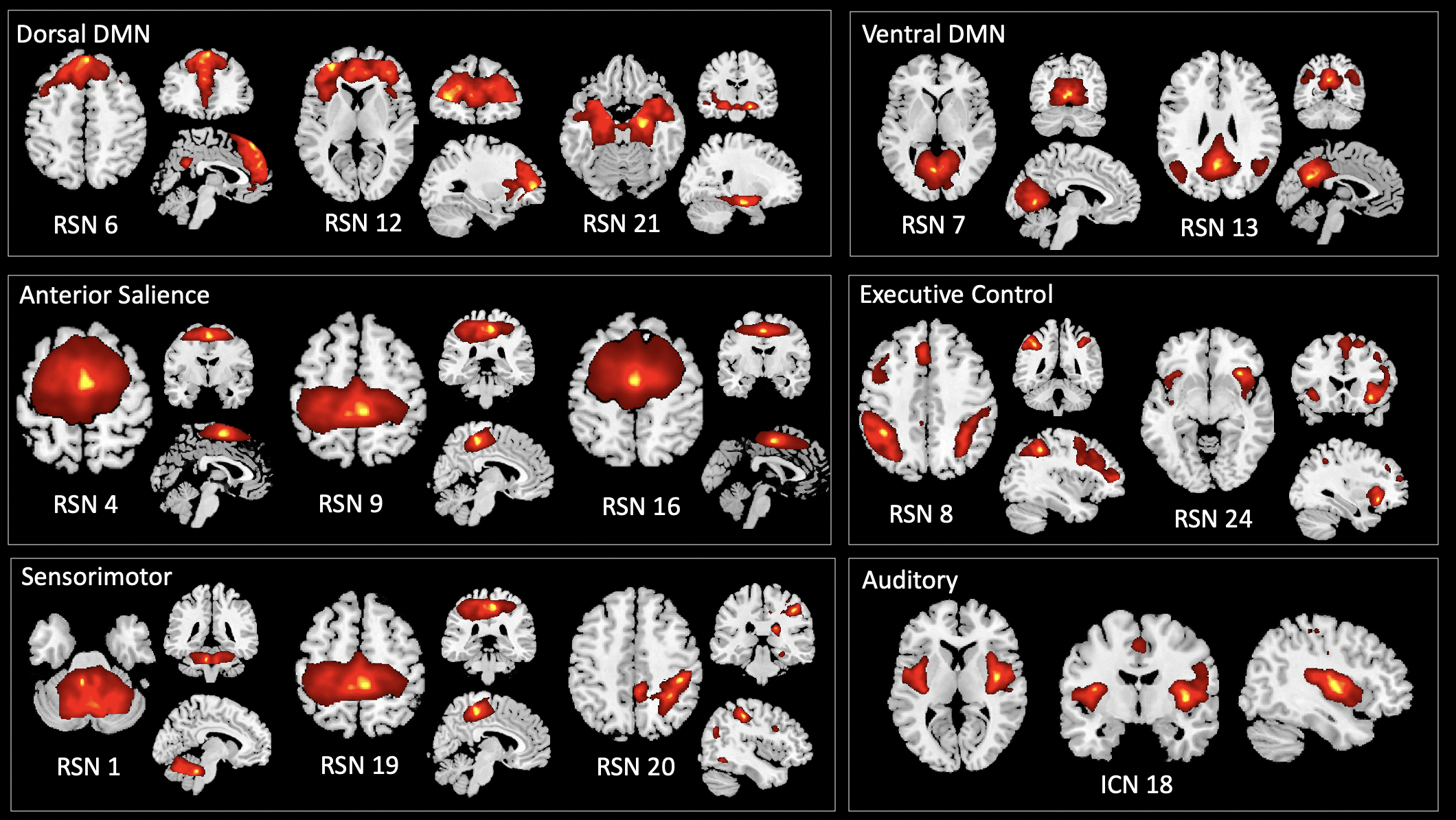
*

*Legend:* Resting state networks derived from spatial ICA are overlaid on a canonical brain thresholded at false discovery rate *p*<0.05. RSN number is displayed beneath, network classification is derived from Shirer *et al.* 32012. DMN: default mode network; RSN: resting state network. Activation thresholded at *T*>20.
