# Supplementary material for: Alcohol use is associated with affective and interoceptive network alterations in bipolar disorder
Source: Brain Behav. 2022 Nov 30;13(1):e2832. doi: 10.1002/brb3.2832 (PMC9847622; doi:10.1002/brb3.2832)
Supplement: Supplementary file 3 — Supplementary Figure 3. Functional activation of the paracingulate gyrus is increased for those with a diagnosis of bipolar disorder who consume alcohol. Legend: BD: participants with a diagnosis of bipolar disorder; HC: healthy controls [file BRB3-13-e2832-s001.docx]

**Supplementary Figure 3.** Functional activation of the paracingulate gyrus is increased for those with a diagnosis of bipolar disorder who consume alcohol.


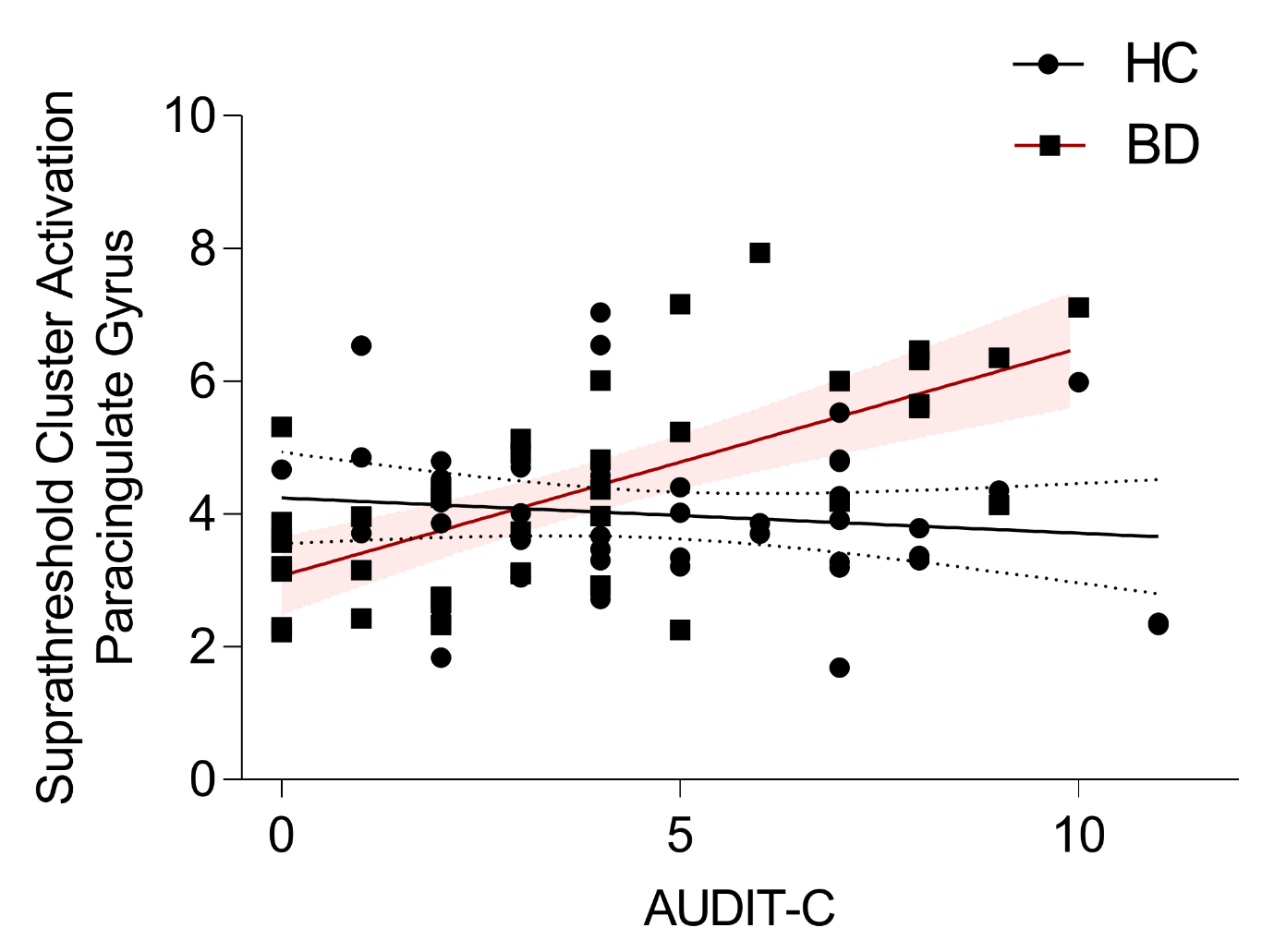


*Legend:* BD: participants with a diagnosis of bipolar disorder; HC: healthy controls
